# Kognition und Kraftfahreignung bei chronischem Schmerzsyndrom

**DOI:** 10.1007/s00115-022-01387-y

**Published:** 2022-09-28

**Authors:** J. Schmidt, M. Weisbrod, M. Fritz, S. Aschenbrenner

**Affiliations:** 1 Abteilung für Klinische Psychologie und Neuropsychologie, SRH Klinikum Karlsbad, Guttmannstr. 1, 76307 Karlsbad-Langensteinbach, Deutschland; 2 Medizinische Fakultät Heidelberg der Universität Heidelberg, Heidelberg, Deutschland; 3 Abteilung für Psychiatrie und Psychotherapie, SRH Klinikum Karlsbad, Karlsbad-Langensteinbach, Deutschland; 4 Klinik für Allgemeine Psychiatrie, Zentrum für Psychosoziale Medizin, Universitätsklinikum Heidelberg, Heidelberg, Deutschland; 5 Abteilung für Neurologie, SRH Klinikum Karlsbad, Karlsbad-Langensteinbach, Deutschland

**Keywords:** Chronischer Schmerz, Kognition, Kraftfahreignung, Fahrverhalten, Neuropsychologische Therapie, Chronic pain, Cognition, Driving ability, Driving behavior, Neuropsychological treatment

## Abstract

Kognitive Auffälligkeiten bei Patienten mit chronischen Schmerzen finden in wissenschaftlichen Untersuchungen zunehmend Beachtung. Die Folgen dieser kognitiven Störungen in Bezug auf die Schmerzbewältigung, die Alltagsgestaltung und die Kraftfahreignung werden in der klinischen Praxis jedoch kaum berücksichtigt, obwohl die Hälfte aller Patienten davon betroffen ist. Die vorliegende Arbeit fasst die aktuelle Studienlage zusammen und diskutiert Möglichkeiten der Integration in die klinische und therapeutische Versorgung.

**Zusatzmaterial online:**

Die Onlineversion dieses Beitrags (10.1007/s00115-022-01387-y) enthält eine zusätzliche Tabelle und zugehörige Literatur. Beitrag und Zusatzmaterial stehen Ihnen auf www.springermedizin.de zur Verfügung. Bitte geben Sie dort den Beitragstitel in die Suche ein, das Zusatzmaterial finden Sie beim Beitrag unter „Ergänzende Inhalte“.

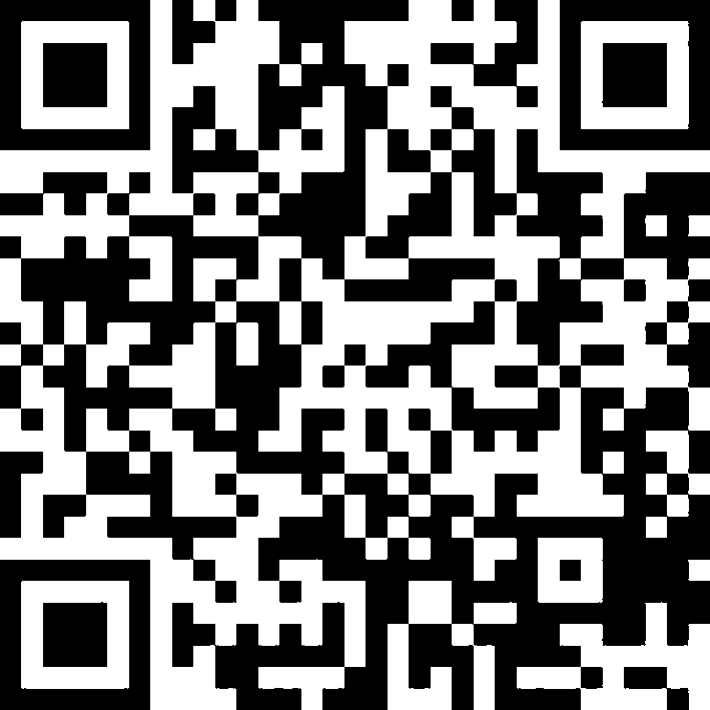

## Abstract (en)

Cognitive impairments in patients with chronic pain are increasingly attracting interest in scientific research. The consequences of these cognitive impairments on coping with pain, everyday life and the driving ability are rarely included in clinical practice although half of all patients are affected. This article summarizes the current research situation and discusses possibilities of the integration in clinical and therapeutic care.

## Hintergrund

Bis zu 30 % der erwachsenen Europäer und US-Amerikaner leiden unter länger andauernden, chronischen Schmerzen [16, 55]. Die Schmerzchronifizierung wird als ein Prozess definiert, bei dem komplexe Wechselwirkungen zwischen biologischen, psychologischen und sozialen Faktoren beteiligt sind [50]. Sie geht mit affektiven Symptomen wie Ängsten, Ärger, Hilflosigkeit, Depressivität und häufigen Schlafstörungen einher. Die höchsten Prävalenzen umfassen Rückenschmerzen (28 %), Kopfschmerzen und Migräne (16 %), Nackenschmerzen (15 %) und Gesichtsschmerz (5 %) [40]. Chronische Schmerzen stellen ein Gesundheitsproblem dar, das maßgeblich die Lebensqualität [16, 25] und somit die soziale und die berufliche Teilhabe beeinflusst [62]. Zu den selbst wahrgenommenen Einschränkungen zählen auch kognitive Störungen, wobei häufig nur die Aufmerksamkeitsstörungen von den Patienten berichtet werden. Betroffen sind jedoch ein breites Spektrum neuropsychologischer Funktionen, wie auch Lern- und Gedächtnisstörungen oder Störungen der Verhaltenssteuerung und Handlungsplanung (Exekutivfunktionen). In verschiedenen Untersuchungen wurden kognitive Störungen bei Patienten mit chronischen Schmerzen festgestellt und auch wiederholt mit der Kraftfahreignung in Verbindung gebracht. Dabei finden sich Hinweise, dass auch Menschen mit chronischen Schmerzen von Einschränkungen der Kraftfahreignung betroffen sein können. Neben kognitiven Störungen bei chronischem Schmerz können auch psychische Begleitsymptome und Komorbiditäten wie Depression und Angst, motorische Einschränkungen, u. a. bedingt durch die schmerzverursachende Grunderkrankung, und pharmakologische Einflüsse wie die Gabe von Opioiden Einfluss auf die Kraftfahreignung haben und kognitive Störungen mit verursachen oder verstärken. Die Einschränkung der Kraftfahreignung stellt nach der International Classification of Functioning, Disability and Health (ICF) eine Barriere bei der Teilhabe am Arbeitsleben dar [62]. Die Diagnostik relevanter neuropsychologischer Maße kann wichtige prognostische Hinweise für den Therapieerfolg liefern und auch individuelle Behandlungsempfehlungen beeinflussen. Dies betrifft sowohl die Therapie chronischer Schmerzen im Allgemeinen als auch die Kraftfahreignung im Speziellen.

## Chronisches Schmerzsyndrom und Kognition

In der Literatur werden Störungen in den Gedächtnis‑, Aufmerksamkeits- und Exekutivfunktionen bei Patienten mit chronischen Schmerzen sowohl in testpsychologischen Untersuchungen als auch in der Selbstauskunft beschrieben [51]. Von den Teilbereichen sind Störungen des Kurzzeitgedächtnisses [15, 46, 68, 85, 117], des Langzeitgedächtnisses [43, 85, 117] sowie des Wiedererkennens [87], Aufmerksamkeitsprozesse, wie die Verarbeitungsgeschwindigkeit [49, 65, 94, 100], die selektive Aufmerksamkeit [15, 26–28, 46, 64], die Daueraufmerksamkeit [43, 84, 103] und exekutive Funktionen, wie das Arbeitsgedächtnis [13, 26–28, 65, 68, 85] und die kognitive Flexibilität [9, 58, 60, 117] betroffen. Allgemein zeigen sich in neuropsychologischen Untersuchungen verlängerte Reaktionszeiten [2, 25, 30, 48, 70]. Menschen mit einer bereits nach einer Schädigung vorhandenen Störung der Exekutiv- oder Gedächtnisfunktionen tragen auch ein höheres Risiko einer Schmerzchronifizierung [10]. Etwa die Hälfte aller Patienten mit chronischen Schmerzen leidet unter einer Störung mindestens einer Modalität kognitiver Funktionen, welche zu Schwierigkeiten in Alltagsfunktionen und psychosozialen Einschränkungen führen [101]. Chronische Schmerzen sind häufig komorbid mit Depression und Angst assoziiert und zwar in einer reziproken, sich gegenseitig verstärkenden Verbindung. Ebenso können bei Depression und Angst neurokognitive Störungen auftreten [23]. Aus diesem Grund wurden kognitive Auffälligkeiten zunächst durch das Vorliegen komorbider depressiver Symptome erklärt [41]. Kognitive Beeinträchtigungen bleiben jedoch auch dann bestehen, wenn das Ausmaß depressiver Symptome statistisch kontrolliert wird [16].

Von Interesse ist es, die ätiologischen Zusammenhänge der Schmerzchronifizierung und kognitiven Störungen zu verstehen. Gehirnareale, die sowohl in Zusammengang mit der Schmerzverarbeitung, der kognitiven und emotionalen Schmerzkontrolle, als auch mit kognitiven Prozessen gebracht werden, umfassen den präfrontalen und den anterioren cingulären Cortex [3, 68, 75]. Es finden sich neuroplastische und hirnmorphologische Veränderungen insbesondere im Bereich des dorsolateralen präfrontalen Cortex [6]. Diese Areale werden ebenfalls mit affektiven Störungen und Angststörungen in Verbindung gebracht [93]. Das macht aus neurobiologischer Perspektive plausibel, weswegen neben kognitiven Defiziten auch Angststörungen und affektive Störungen im Verlauf auftreten.

Auch ein reduziertes Volumen des Hippokampus, der mit Gedächtnisprozessen assoziiert wird, wurde bei Menschen mit chronischen oder akuten Schmerzen gefunden [6]. Chronische Schmerzen werden auch mit einer Volumenminderung der grauen Substanz in Zusammenhang gebracht [6].

Kognitive Einschränkungen bei Patienten mit chronischen Schmerzen werden bei allen Schmerzformen (nozizeptiv, neurogen, psychogen, gemischt) und Lokalisationen der Schmerzen berichtet. Sie wurden bei Patienten mit komplexem regionalem Schmerzsyndrom (CRPS), Fibromyalgie, rheumatoider Arthritis, muskuloskelettaler Erkrankung und Migräne untersucht [26, 37, 47]. Dabei nimmt die spezielle Schmerzform Einfluss auf das Ausmaß und die Art der vorliegenden kognitiven Störung und erhöht deren Auftretenswahrscheinlichkeit. Es ist also durchaus sinnvoll, den einzelnen Schmerzformen genauere Beachtung zu schenken. So zeigen sich bei Menschen mit chronischen Schmerzen, deren Schmerz mehrere Körperbereiche betrifft, so wie bei der Fibromyalgie, deutlichere Beeinträchtigungen von Konzentration und Gedächtnis [43, 45, 68, 87, 97]. Dieser Unterschied zeigte sich im Vergleich zu Menschen mit neuropathischen Schmerzen und zu gemischten Schmerzen und blieb trotz Berücksichtigung von komorbiden affektiven und Angstsymptomen bestehen [97]. Menschen mit Fibromyalgie klagen auch stärker über Gedächtnisstörungen und depressive Symptome als Menschen mit anderen chronischen Schmerzformen [24, 87, 104]. Bei generalisiertem und neuropathischem Schmerz wird ein größerer Effekt auf die Kognition angenommen als bei lokalem Schmerz [64]. Bei Menschen mit rheumatoider Arthritis werden ebenfalls vermehrt depressive Symptome berichtet [18]. Ebenfalls ergeben sich deutliche Einschränkungen der Daueraufmerksamkeit und des Arbeitsgedächtnisses [26]. Menschen mit chronischer Migräne weisen Störungen des Gedächtnisses, insbesondere während den Attacken und Störungen der Exekutivfunktionen [37, 42, 72] auf. Bei Menschen mit Schädigung nach Schleudertraumata finden sich Einschränkungen der selektiven Aufmerksamkeit, des verbalen Gedächtnisses, des Arbeitsgedächtnisses und der verbalen Flüssigkeit [2, 15], bei Rückenschmerzpatienten eine reduzierte Verarbeitungsgeschwindigkeit, Störungen bei dem Abruf verbaler Information sowie der kognitiven Umstellungsfähigkeit [102, 117]. Neben der Schmerzform muss ebenfalls die Dauer der Schmerzerkrankung, die Stärke der Schmerzen, als individuelles Maß, sowie das Alter der Schmerzpatienten berücksichtigt werden. Bei Menschen mit neuropathischen Schmerzen ergeben sich Hinweise, dass die kognitiven Beeinträchtigungen zunächst mit der Dauer der Schmerzen zunehmen, sich jedoch im weiteren Verlauf wieder verbessern [82]. Dieser U‑förmige Verlauf kommt möglicherweise durch eine zunehmend verbesserte Auseinandersetzung mit dem Schmerz und Akzeptanz zustande, sodass weniger kognitive Kapazitäten gebunden werden. Neben Studien die einen Zusammenhang zwischen kognitiven Störungen und Schmerzdauer annehmen [5, 82, 115], werden ebenfalls keine Zusammenhänge oder inverse Zusammenhänge berichtet [30]. Menschen, die eine größere Schmerzstärke berichteten, erzielten schlechtere Ergebnisse in kognitiven Untersuchungen [1, 46, 59, 92, 96]. Möglicherweise führt auch hier die Schmerzstärke zu einer stärkeren kognitiven Beschäftigung mit dem Schmerz, sodass in relevantem Ausmaß kognitive Kapazitäten gebunden werden. Kognitive Einschränkungen wurden ebenfalls unabhängig vom Alter der Schmerzpatienten nachgewiesen [74]. Eine aufschlussreiche Studie differenziert zwischen dem Alter der Patienten mit chronischen Schmerzen und der Schmerzstärke. So fanden sich negative Zusammenhänge zwischen der Schmerzstärke und Exekutiv- und Gedächtnisfunktionen bei jüngeren Patienten, während keine Zusammenhänge zwischen Gedächtnis und Schmerzstärke und sogar positive Zusammenhänge zu Exekutivfunktionen bei älteren Patienten gefunden wurden [86]. Auch zeigen ältere Menschen mit Schmerzen (65–75) eine geringere Tendenz zur Katastrophisierung und eine bessere Schmerzakzeptanz im Vergleich zu Schmerzpatienten mittleren (40–64) oder jüngeren (18–39) Alters [78].

Zuletzt soll auf den Einfluss von Medikation auf das Vorhandensein kognitiver Störungen eingegangen werden. Bei der medikamentösen Behandlung chronischer Schmerzen können heterogene Effekte erwartet werden. So kann die Ein‑, Auf- und Umstellung von Medikamenten relevante kognitive Leistungsbeeinträchtigungen, aber auch eine Verbesserung der Kognition zur Folge haben. Eine besondere Bedeutung nehmen Opioide ein, die mit ihrer Wirkung auf das zentrale Nervensystem eine maßgebliche Rolle in der Schmerztherapie haben. Dabei wurden insbesondere Einschränkungen im Arbeitsgedächtnis, der Aufmerksamkeit (insbesondere komplexe Aufmerksamkeitsprozesse) und der Verarbeitungsgeschwindigkeit gefunden [33, 102]. Dagegen stehen Studien, die keinen Effekt oder sogar eine Verbesserung der Kognition durch Opioide belegen [54, 71, 110]. Ebenfalls sollte die Stabilität der Einstellung berücksichtigt werden. Einflüsse von Opioiden auf die Kognition sind nachweislich nach einer Woche der Medikation nicht mehr vorhanden [19, 39]. Dagegen führt der Gebrauch anderer psychoaktiver Substanzen wie Benzodiazepinen zu kognitiven Einschränkungen [99].

Neben hirnmorphologischen Veränderungen gibt es diverse neuropsychologische Erklärungsansätze für die möglichen Ursachen kognitiver Störungen. In einem gängigen neurokognitiven Modell [66] wird angenommen, dass nur eine begrenzte Kapazität von Aufmerksamkeitsleistungen besteht. Chronischer Schmerz, der mit einer permanenten nozozeptiven Stimulation einhergeht, führt zu chronischen Begrenzungen gar Überlastungen der Kapazität und zu einer Verlangsamung oder dem Abbruch von Prozessen. Es wird postuliert, dass Schmerz, im Sinne einer Störvariable, Aufmerksamkeitsprozesse beansprucht und andere Informationen in den Hintergrund rücken [31]. Auf diese Weise findet eine Priorisierung der Schmerzverarbeitung statt, um die Schmerzursache schnellstmöglich zu finden und zu beseitigen, entwicklungsgeschichtlich sinnvoll, um die Überlebenswahrscheinlichkeit zu erhöhen. Durch den Versuch, Schmerzen zu bewältigen, verändert sich etwa die Fähigkeit, komplexe kognitive Aufgaben zu bewältigen [31]. Da Schmerz insbesondere komplexe Aufmerksamkeits- und Exekutivfunktionen bindet, kommt es zu einer allgemeinen Interferenz mit der kognitiven Kontrolle [77]. Für die Implementierung von Copingstrategien, wie der adäquaten Regulation von Emotionen, der Etablierung kontrollierender Gedanken und der Anpassung der Zielsetzung [22], werden intakte Exekutivfunktionen wie Inhibition und kognitive Flexibilität benötigt. Störungen der Exekutivfunktionen führen wiederum dazu, dass der Einsatz von Schmerzbewältigungsstrategien eingeschränkt sein könnte [20]. Eine weitere mögliche Erklärung für die Defizite in der Kontrolle von Aufmerksamkeitsprozessen könnte in einer reduzierten kognitiven Verarbeitungsgeschwindigkeit liegen [84, 114]. Die Theorie konkurriert zu der allgemeinen Annahme, dass die kognitiven Störungen allein durch exekutive Störungen herbeigeführt werden. Daneben werden Defizite im Arbeitsgedächtnis diskutiert [13].

## Chronisches Schmerzsyndrom und Fahrverhalten

Das adäquate Führen von Fahrzeugen ist eine komplexe Aufgabe und setzt spezifische kognitive Fähigkeiten voraus [35], die in einem hohen Maß von Übung und Erfahrung abhängen [89]. Erforderlich sind Wahrnehmungs- und Aufmerksamkeitsfunktionen sowie die Fähigkeit zur schnellen Entscheidungsfindung. Der Einfluss von Schmerzen sowohl auf die körperliche Funktionsfähigkeit als auch auf die Kognition könnte einen Einfluss auf die Wahrscheinlichkeit von Unfallgeschehen haben [111]. Bei Menschen mit chronischen Schmerzen zeigen sich in mehreren für die Fahrkompetenz als relevant erachteten Fähigkeiten Einschränkungen, wie den Aufmerksamkeits- und Exekutivfunktionen [26, 46, 49, 103]. Einen Überblick über die erforderlichen operationalen, taktischen und strategischen Kompetenzen, die zum Führen eines Fahrzeuges benötigt werden, liefert das Mehrebenenmodell von Michon [73]. In diesem Modell werden die Anforderungen an das Fahren in drei Ebenen untergliedert. Die strategische Ebene umfasst Anforderungen der Navigation vor und während der Fahrt, wie die Auswahl einer bestimmten Route. Die taktische Ebene umfasst die Führung des Kraftfahrzeugs und beinhaltet das Ausführen von Fahrmanövern oder das Überholen und Abbiegen. Die operationale Ebene umfasst das Beschleunigen, Bremsen oder Lenken des Kraftfahrzeugs. Dieses Modell wird häufig durch das Skill-Rules-Knowledge-Modell (SRK-Modell) ergänzt, welches eine wissensbasierte, regelbasierte und fertigkeitsbasierte Ebene einschließt [29]. Ein Verhalten in unbekannten Situationen umfasst die wissensbasierte Ebene, während auf der regelbasierten Ebene bei Entscheidungen auf Wissen aus früheren Erfahrungen zurückgegriffen werden kann. Routiniert wiederkehrende und erlernte Handlungen werden fertigkeitsbasiert getroffen. Es gibt nur wenige Studien, die sich mit den Einflüssen chronischer Schmerzen auf die Kraftfahreignung beschäftigen. In den meisten Studien wird der Einfluss von Opioiden auf die Kraftfahreignung untersucht (z. B. [99, 100]). Vieles spricht dafür, dass chronischer Schmerz das Fahrverhalten stärker beeinflusst als die therapeutische Gabe von Opioiden [36]. Studien belegen, dass Patienten mit chronischen Schmerzen Einschränkungen beim Autofahren erleben [12, 34, 52, 56, 88, 98, 105, 106], ein höheres Risiko tragen, in Autounfälle verwickelt zu werden [11, 34, 38, 63, 69, 95, 112], höhere Abweichungen von der Fahrbahnmittellinie aufweisen [113] und verlängerte Reaktionszeiten aufzeigen [80, 81, 109]. Dabei entsprachen Abweichungen, jenen, die bei gesunden Probanden mit einer Blutalkoholkonzentration von 0,8 Promille gefunden wurden [67]. Darunter wird die Wahrscheinlichkeit, einen Unfall zu begehen, als dreimal höher eingeschätzt [14]. Zudem werden Verkehrsschilder in standardisierten Teststrecken übersehen [80]. Eine aktuelle Übersichtsarbeit ordnet die gefundenen Einschränkungen den operationalen, taktischen und strategischen Kompetenzen des Mehrebenenmodells zu und zeigt, dass Menschen mit chronischen Schmerzen zu kompensatorischen Strategien greifen, um Autounfälle zu vermeiden [112]. So vermeiden Menschen mit chronischen Schmerzen häufig das Autofahren gänzlich oder das Fahren unter bestimmten Bedingungen wie Nachtfahrten oder Fahrten bei schlechtem Wetter [89, 106]. Allerdings finden sich auch Studien, die keine Effekte auf die Kraftfahreignung zeigen [12, 21, 57, 83, 102, 107, 108]. Einen ausführlichen Überblick über die Studienlage gibt eTabelle 1 (siehe elektronisches Zusatzmaterial).

## Chronisches Schmerzsyndrom und Kraftfahreignung

Die Kraftfahreignung und Voraussetzungen der Teilnahme am Straßenverkehr sind in Deutschland mit dem Straßenverkehrsgesetz (StVG) gesetzlich geregelt. Die Eignung zum Führen von Fahrzeugen ist bei Menschen mit chronischen Schmerzen nicht prinzipiell ausgeschlossen. Da die Kraftfahreignung ein wichtiges Gut zur Teilhabe darstellt, sind die Bedingungen für den Erhalt der Kraftfahreignung zu definieren. Zur Beurteilung der Fahreignung werden die Begutachtungslinien zur Kraftfahrereignung der Bundesanstalt für Straßenwesen (BASt) herangezogen [44]. Dort sind ebenfalls spezifische Krankheiten unter medizinischer und psychologischer Betrachtung aufgeführt. Schmerzerkrankungen im Speziellen werden nicht berücksichtigt. Im speziellen Teil der Begutachtungsleitlinien werden psychische Störungen, Einschränkungen des Seh- und Hörvermögens, Bewegungsbehinderungen, Störungen des Gleichgewichtssinnes (Schwindel, Migräneschwindel) und spezifische somatische Erkrankungen aufgeführt, welche zur Beurteilung im Einzelfall herangezogen werden können. Ein verkehrsmedizinisches Gutachten kann von der Fahrerlaubnisbehörde angeordnet werden, wenn ihr Zweifel an der Fahreignung zur Kenntnis kommen. Ärzte an anerkannten Begutachtungsstellen und Fachärzte mit Zusatzbezeichnung Verkehrsmedizin dürfen diese Gutachten erstellen. Da jeder Verkehrsteilnehmer eine Vorsorgepflicht hat, andere Verkehrsteilnehmer nicht zu gefährden, kann auch eine selbstinitiierte Untersuchung der körperlichen und psychischen Eignung erfolgen, welche um eine praktische Fahrverhaltensprobe ergänzt werden kann. Zur Erhebung der Fahrkompetenz werden überwiegend verkehrspsychologische Testverfahren, praktische Fahrverhaltensproben und selten auch Untersuchungen am Fahrsimulator herangezogen [17, 44, 91]. Der aufbereiteten Literatur zufolge sollte den Bereichen Kognition, Psyche, Somatik und Medikation bei chronischem Schmerzsyndrom genauere Betrachtung zuteilwerden. Einen Überblick über die rechtlichen Aspekte der Fahreignung im Allgemeinen gibt Tab. 1.

**Table 1:** 

Regelung durch	Inhalt
Straßenverkehrsgesetz (StVG), § 2, Absatz 4	Fahrerlaubnis und Führerschein: Erfüllung notwendiger körperlichen, geistiger Anforderungen. Fehlen erheblicher/wiederholter Verstöße gegen StVG/allgemeine Strafgesetze
Fahrerlaubnis-Verordnung (FeV), § 2	Eingeschränkte Zulassung. Pflicht der eigenen Vorsorge, insbesondere bei körperlicher/geistiger Beeinträchtigung
Begutachtungsleitlinien Bundesanstalt für Straßenwesen (BASt)	Aufführung spezieller körperlicher, psychischer Erkrankungen. Bezug zu der Einnahme von Medikation
Bürgerliches Gesetzbuch (BGB)	Aufklärungs- und Dokumentationspflicht, Zivil- oder strafrechtliche Haftungspflicht (§ 611, § 280, § 253 BGB)
Strafgesetzbuch (StGB)	Verstoß gegen Vorsorgenpflicht: Geld- und/oder Freiheitstrafen (§ 316 StGB). Auswirkungen auf Versicherungsschutz. § 34 Rechtfertigender Notstand

## Kognition

Aus neuropsychologischer Sicht sind insbesondere die folgenden Fähigkeiten verkehrs- und sicherheitsrelevant: allgemeine Reaktionsbereitschaft, Daueraufmerksamkeit, räumliche Aufmerksamkeitsausrichtung, Aufmerksamkeitsteilung, fokussierte Aufmerksamkeit und Flexibilität [91], wenngleich diese noch keinen Eingang in die Gesetzgebung (Fahrerlaubnis-Verordnung, FeV) gefunden haben. Wie beschrieben, sind Störungen der Aufmerksamkeit, des Gedächtnisses und der Exekutivfunktionen bei chronischem Schmerzsyndrom häufig [51]. Bei den Gedächtnisfunktionen sind Störungen des Kurzzeitgedächtnisses [15, 46, 68, 85, 117], des Langzeitgedächtnisses [43, 85, 117] sowie des Wiedererkennens [87] bekannt. Störungen der Aufmerksamkeit umfassen die Verarbeitungsgeschwindigkeit [49, 65, 94, 100], die selektive Aufmerksamkeit [15, 26–28, 46, 64] und die Daueraufmerksamkeit [43, 84, 103]. Exekutivfunktionsstörungen bei chronischem Schmerzsyndrom beinhalten das Arbeitsgedächtnis [13, 26–28, 65, 68, 85] und die kognitive Flexibilität [9, 58, 60, 117]. Zusätzlich sind verlängerte Reaktionszeiten zu erwarten [2, 25, 30, 48, 70]. Die Beurteilungskategorien der neuropsychologischen Untersuchung werden entsprechend der Anlage 5 der FeV ausgewählt. Dies ist unabhängig von psychischen Komorbiditäten zu berücksichtigen. Mögliche Einschränkungen sollen mit dafür geeigneten, objektivierbaren und psychologischen Testverfahren untersucht werden. Eine allgemein gültige Empfehlung bestimmter Testverfahren und der Anzahl einzusetzender Verfahren liegt aktuell nicht vor [79]. Eine adäquate Erfassung der Leistungsbereiche ermöglicht beispielsweise das Wiener Testsystem (Schuhfried), die Testbatterie zur Aufmerksamkeitsprüfung in der Version Mobilität (Psytest) oder die ART2020 (Schuhfried) [79]. Bei der Interpretation sind altersunabhängige Normen, wie in der Begutachtungsleitlinie zur Kraftfahreignung ausdrücklich vorgesehen, zu berücksichtigen. Zweifel an der Kraftfahreignung bestehen, wenn die Mindestanforderungen (Prozentrang > 16) nicht erfüllt sind. Eine Fahrverhaltensprobe kann bei objektivierten Leistungsdefiziten in Einklang mit den Empfehlungen der Begutachtungslinien zur Kraftfahrteignung erwogen werden [79]. Als Ergänzung können Fahrsimulatoren sowohl zur Fahreignungsprüfung als auch beim Training eingesetzt werden [7]. Trotz nachgewiesener Evidenz werden Fahrsimulatoren noch nicht standardmäßig eingesetzt [61]. Tab. 2 gibt einen Überblick über mögliche neuropsychologischen Testverfahren aus dem Wiener Testsystem, die bei Menschen mit chronischem Schmerzsyndrom aus Sicht der Autoren eine fachlich sinnvolle Diagnostik erlauben.

**Table 2:** 

Testverfahren	Geprüfte Funktion	Testform
Reaktionstest (RT)	Reaktionsfähigkeit	S3
Cognitrone (COG)	Konzentration	S11
Wahrnehmungs- und Aufmerksamkeitsfunktionen – Batterie: Geteilte Aufmerksamkeit (WAF‑G)	Geteilte Aufmerksamkeit	S2
Adaptiver Tachistoskopischer Verkehrsauffassungs-Test (ATAVT)	Aufmerksamkeitsleistung, Beobachtungsfähigkeit	S1
Response Inhibition (INHIB)	Inhibitionsfähigkeit	S13
N-BACK Verbal (NBV)	Arbeitsgedächtnis	S1
Linienverfolgungstest (LVT)	Orientierungsleistung	S3
Determinationstest (DT)	Belastbarkeit	S1

## Psyche

Kognitive Beeinträchtigungen, sowohl in subjektivem als auch in objektivem Ausmaß, finden sich in unterschiedlicher Ausprägung bei einer Vielzahl psychischer Störungen. Kognitive Einschränkungen können nach Remission der psychischen Erkrankung abklingen, jedoch auch überdauernder Natur, im Sinne einer Chronifizierung, sein. Psychische Erkrankungen können komorbid zu chronischen Schmerzen auftreten. Primäre oder komorbide psychische Erkrankungen sind affektive Störungen, Suchterkrankungen, Schlafstörungen oder Persönlichkeitsstörungen. Zu beachten ist, dass die psychische Leistungsfähigkeit keinen stabilen Wert darstellt, sondern auch vorübergehenden Einschränkungen unterliegen kann (z. B. Müdigkeit, Schmerzen).

Eine Beurteilung der Fahreignung sollte auch hier anhand objektivierbarer und standardisierter Leistungsdiagnostik erfolgen.

## Somatik

Somatische Beschwerden sind vollumfänglich in Bezug auf die Kraftfahreignung zu berücksichtigen und fachlich zu beurteilen. Im Einzelfall differenziert zu betrachten ist Schwindel. Hauptsächlich beachtet werden motorische Einschränkungen. Auswirkungen operativer Eingriffe bspw. an der Wirbelsäule sind in die Beurteilung zu integrieren. Verletzungen der Halswirbelsäule können in Zusammenhang mit Schwindel stehen. Bewegungseinschränkungen sind häufige Begleiterscheinungen bei Menschen mit chronischem Schmerzsyndrom. Auch die somatischen Störungen der schmerzverursachenden Grunderkrankungen (z. B. Lähmungen bei neurologischen Erkrankungen mit chronischem neuropathischem Schmerz) oder Gelenk- und Wirbelsäulenbewegungseinschränkungen bei rheumatologischen Erkrankungen und Wirbelsäulenleiden mit chronischem Schmerz sind zu berücksichtigen. In Anhang B der Begutachtungsleitlinien der BASt wird das Anbringen geeigneter Einrichtungen am Fahrzeug, der Ersatz fehlender Gliedmaßen mittels künstlicher Glieder, eine Begleitung oder das Tragen spezieller Abzeichen und Kennzeichen aufgeführt.

## Medikation

In den Begutachtungsleitlinien zur Kraftfahrereignung der BASt wird Menschen, die Betäubungsmittel einnehmen oder von solchen abhängig sind, eine Fahreignung aberkannt. Die Einnahme eines Medikaments im konkreten Krankheitsfall, welche demnach bestimmungsgemäß erfolgt, ist davon ausgenommen. Bei der Behandlung chronischer Schmerzen sind neben Schmerzmedikamenten (insbesondere Opioide) auch Schlaf- und Beruhigungsmittel, Antikonvulsiva, Neuroleptika und Antidepressiva zu berücksichtigen. Insbesondere bei neuropathischen Schmerzen ist auch der Einfluss von Koanalgetika wie Amitritylin, Pregabalin, Gabapentin auf die Kognition zu berücksichtigen. Bei Eindosierung einer solchen Substanzklasse und/oder Dosiserhöhung werden relevante Leistungsbeeinträchtigungen erwartet. Erwogen werden kann eine Fahrpause von 1 bis 2 Wochen. Eine differenzierte Betrachtung sollte erfolgen, da die Einnahme ebenfalls eine Symptomverbesserung der Grunderkrankung zur Folge haben kann. Zur Orientierung kann auf die Kategorisierung für psychotrope Substanzen des International Council on Alcohol, Drugs and Traffic Safety (ICADTS) zurückgegriffen werden [53]. Die dreistufige Kategorisierung zieht Blutalkoholäquivalenzdosen zum Vergleich heran. Unterschieden werden Medikamente der Kategorie I, ohne relevanten Einfluss, Medikamente der Kategorie II mit geringem oder moderatem Einfluss auf die Fahreignung sowie jene mit Gefahrenpotenzial (Kategorie III). Tab. 3 gibt einen Überblick über die relevanten Medikamente zur Behandlung des chronischen Schmerzsyndroms.

**Table 3:** 

Substanzname	Beispiele	Kategorie
Analgetika	Opioide (Morphin, Hydromorphon, Oxycodon, Codein, Fentanyl, Tilidin, Tramadol)	II–III
	Acetylsalicylsäure (Kombination)	II
	Paracetamol	II
	Triptane (Sumatriptan, Naratriptan, Rizatriptan)	II
Antiinflammatorika und Antirheumatika	Diclofenac, Ibuprofen, Naproxen	I
Muskelrelaxanzien	Tetrazepam	II
Anästhetika	Opioide (Fentanyl)	III
Antiepilektika	Lamotrigin, Gabapentin, Levetiracetam	II
Neuroleptika	Promazin, Haloperidol, Melperon, Pipamperon, Clozapin, Olanzapin, Quetiapin	II
Lithium	Lithium	II
Anxiolytika	Benzodiazepine (Diazepam, Oxazepam, Lorazepam, Bromazepam)	III
Antidepressiva	Fluoxetin, Citalopram, Paroxetin, Sertalin, Escitalopram	I
	Imipramin, Opipramol	II
	Venlafaxin, Milnacipran, Trazodon, Mirtazapin	II–III
	Trimipramin, Amitriptylin, Doxepin	III
Andere – zentral wirksam	Bupropion, Naltrexon, Methadon	II

Kategorie I (Äquivalenzdosis zu Blutalkoholgehalt < 0,05 %), Kategorie II (Äquivalenzdosis zu Blutalkoholgehalt 0,05–0,08 %), Kategorie III (Äquivalenzdosis zu Blutalkoholgehalt > 0,08 %)

## Integration in die klinische und therapeutische Versorgung

Das Vorhandensein kognitiver und psychischer Störungen muss bei Menschen mit chronischem Schmerzsyndrom in der Praxis immer mitbedacht und berücksichtigt werden. Beeinflusst werden sowohl die subjektive Lebenszufriedenheit, der Verlauf der Erkrankung als auch Möglichkeiten der sozialen und beruflichen Integration. Dabei ist natürlich die Kraftfahreignung maßgeblich zu erwähnen. Eine frühzeitige Diagnostik kognitiver und psychischer Störungen bei Patienten mit chronischen Schmerzen ist für die klinische Versorgung sinnvoll. Die Diagnostik sollte aus Sicht der Autoren die neuropsychologische Abklärung und eine Einschätzung der Kraftfahreignung ermöglichen. Als psychologische Basisdiagnostik werden Screenings für Depression und Angst sowie die Erfassung von Schmerzcopingstrategien und ein perspektivischer Zugang zur professionellen psychotherapeutischen Unterstützung empfohlen [76].

Im Folgenden soll näher auf die Möglichkeiten der neuropsychologischen Therapie eingegangen werden. Eine Restitution der Leistungen der Aufmerksamkeits- und Exekutivfunktionen im Rahmen kognitiver Trainings zur Wiederherstellung der Fahreignung sollte angestrebt werden. Ebenfalls, und als effektiver eingeschätzt, kann ein Fahrsimulatortraining genutzt werden, um das Fahren in verschiedenen Szenarien zu erproben [7]. Orientiert am Mehrebenenmodell gilt dieses als effektive Intervention bei Einschränkungen auf der taktischen und operationalen Ebene [7]. Dieses sollte, in Übereinstimmung mit den Empfehlungen der Begutachtungsleitlinien, um eine Fahrverhaltensprobe bzw. Fahrstunden bei einer Fahrschule ergänzt werden. Ein solches Vorgehen wäre wünschenswert, ist jedoch durch die ambulante Versorgung nicht zugelassen, da es aktuell keinen Rahmen für eine kassenärztliche Behandlung gibt. Institutionell wäre dies ein sozialrechtlicher Auftrag an Rehabilitationskliniken, dort stehen kognitive Einschränkungen und die Auswirkungen auf die Fahreignung jedoch nur bei Kliniken mit neurologischem Schwerpunkt im Fokus. Neben restitutiven Ansätzen sind kompensatorische Ansätze zu erwähnen. Darunter können technische Kompensation (technische Hilfsmittel z. B. Fahrzeugassistenzsysteme), sozialorganisatorische Kompensation (Unterstützung durch Mitfahrer, Anpassung der Ziele), verhältnismäßige Kompensation (wie z. B. weniger fahren) und kognitive Kompensation (z. B. bewusste Erhöhung der Anstrengung) zusammengefasst werden [32]. Da eine medikamentöse Behandlung ebenfalls zu einer Verbesserung der Kognition beitragen kann [54, 71, 110], fällt darunter auch die medizinische Kompensation. Eine Aufklärung des Patienten über die bestehenden Möglichkeiten wird empfohlen. Im psychotherapeutischen Setting der Schmerztherapie ist bei Hinweisen auf kognitive Störungen eine Anpassung an das kognitive Niveau des Patienten zu empfehlen. Hierbei können integrative und umfassende Ansätze der individualisierten Neuropsychotherapie (INPT) hilfreich sein [8]. Bei der Schmerztherapie kommen psychologische Techniken wie Ablenken, Achtsamkeit und Meditation zum Einsatz. Schmerzreduktion wird dabei durch hemmende Top-down-Prozesse multipler Gehirnregionen erreicht [4, 90, 116]. Da möglicherweise auch eine Verbesserung kognitiver Störungen durch eine verbesserte Akzeptanz der Schmerzsymptomatik erreicht werden kann, können auch kognitive Verfahren als Analgesie mitberücksichtigt werden.

## Fazit

Bei Patienten mit chronischen Schmerzen können kognitive Beeinträchtigungen vorhanden sein und wichtige prognostische Hinweise für den Therapieerfolg und Behandlungsempfehlungen liefern. Da chronischer Schmerz möglicherweise zu der eingeschränkten Fähigkeit führt, adäquat auf relevante Stimuli im Straßenverkehr zu reagieren, muss auch immer die Kraftfahreignung mitbedacht werden. Die Diagnostik und Mitbehandlung kognitiver Einschränkungen bei der Behandlung chronischer Schmerzen, beispielsweise im Rahmen einer multimodalen Schmerztherapie, ist unbedingt zu empfehlen.

## Supplementary Information






## References

[1] Abeare CA, Cohen JL, Axelrod BN (2010). Pain, executive functioning, and affect in patients with rheumatoid arthritis. Clin J Pain.

[2] Antepohl W, Kiviloog L, Andersson J (2003). Cognitive impairment in patients with chronic whiplash-associated disorder—a matched control study. NeuroRehabilitation.

[3] Apkarian AV, Baliki MN, Geha PY (2009). Towards a theory of chronic pain. Prog Neurobiol.

[4] Apkarian AV, Bushnell MC, Treede RD, Zubieta JK (2005). Human brain mechanisms of pain perception and regulation in health and disease. Eur J Pain.

[5] Apkarian AV, Sosa Y, Krauss BR (2004). Chronic pain patients are impaired on an emotional decision-making task. Pain.

[6] Apkarian AV, Sosa Y, Sonty S, Levy RM, Harden RN, Parrish TB (2004). Chronic back pain is associated with decreased prefrontal and thalamic gray matter density. J Neurosci.

[7] Aschenbrenner S, Schale A, Weisbrod M (2020). Wiedererlangung der Fahrkompetenz. Behandlungsziel bei psychischen Erkrankungen. Nervenheilkunde.

[8] Aschenbrenner S, Schilling T, Grossmann J, Heck T, Bossert M (2020). Psychische Störungen nach erworbener ZNS-Schädigung. Psych Up2date.

[9] Attal N, Masselin-Dubois A, Martinez V (2014). Does cognitive functioning predict chronic pain? Results from a prospective surgical cohort. Brain.

[10] Attal N, Masselin-Dubois A, Martinez V, Jayr C, Albi A, Fermanian J, Bouhassira D, Baudic S (2014). Does cognitive functioning predict chronic pain? Results from a prospective surgical cohort. Brain.

[11] Bell T, Pope C, Fazeli P, Crowe M, Ball K (2020). The association of persistent low back pain with older adult falls and collisions: a longitudinal analysis. J Appl Gerontol.

[12] Benyamina Douma N, Côté C, Lacasse A (2018). Occupational and ergonomic factors associated with low back pain among car-patrol police officers. Clin J Pain.

[13] Berryman C, Stanton TR, Bowering KJ (2013). Evidence for working & memory deficits in chronic pain: a systematic review and meta-analysis. Pain.

[14] Borkenstein RF, Crowther FR, Shumate RP, Ziel WB, Zylman R (1964). The role of the drinking driver in traffic accidents.

[15] Bosma FK, Kessels RP (2002). Cognitive impairments, psychological dysfunction, and coping styles in patients with chronic whiplash syndrome. Cogn Behav Neurol.

[16] Breivik H, Collett B, Ventafridda V, Cohen R, Gallacher D (2006). Survey of chronic pain in Europe: prevalence, impact on daily life, and treatment. Eur J Pain.

[17] Brenner-Hartmann J (2002). Durchführung standardisierter Fahrverhaltensbeobachtungen im Rahmen der medizinisch-psychologischen Untersuchung (MPU).

[18] Brown SC, Glass JM, Park DC (2002). The relationship of pain and depression to cognitive function in rheumatoid arthritis patients. Pain.

[19] Bruera E, Macmillan K, Hanson J, MacDonald RN (1989). The cognitive effects of the administration of narcotic analgesics in patients with cancer pain. Pain.

[20] Bushnell MC, Ceko M, Low LA (2013). Cognitive and emotional control of pain and its disruption in chronic pain. Nat Rev Neurosci.

[21] Byas-Smith MG, Chapman SL, Reed B, Cotsonis G (2005). The effect of opioids on driving and psychomotor performance in patients with chronic pain. Clin J Pain.

[22] Caes L, Dick B, Duncan C, Allan J (2021). The cyclical relation between chronic pain, executive functioning, emotional regulation, and self-management. J Pediatr Psychol.

[23] Castaneda AE, Tuulio-Henriksson A, Marttunen M, Suvisaari J, Lonnqvist J (2008). A review on cognitive impairments in depressive and anxiety disorders with a focus on young adults. J Affect Disord.

[24] Castel A, Cascón-Pereira R, Boada S (2021). Memory complaints and cognitive performance in fibromyalgia and chronic pain: the key role of depression. Scand J Psychol.

[25] Coppieters I, Ickmans K, Cagnie B (2015). Cognitive performance is related to central sensitization and health-related quality of life in patients with chronic whiplash-associated disorders and fibromyalgia. Pain Physician.

[26] Dick B, Eccleston C, Crombez G (2002). Attentional functioning in fibromyalgia, rheumatoid arthritis, and musculoskeletal pain patients. Arthritis Care Res.

[27] Dick BD, Rashiq S (2007). Disruption of attention and working memory traces in individuals with chronic pain. Anesth Analg.

[28] Dick BD, Verrier MJ, Harker KT (2008). Disruption of cognitive function in fibromyalgia syndrome. Pain.

[29] Donges E, Winner H, Hakuli S, Lotz F, Singer C (2015). Fahrerverhaltensmodelle. Handbuch Fahrerassistenzsysteme. Grundlagen, Komponenten und Systeme für aktive Sicherheit und Komfort.

[30] Eccleston C (1994). Chronic pain and attention: a cognitive approach. Br J Clin Psychol.

[31] Eccleston C, Crombez G (1999). Pain demands attention: a cognitive-affective model of the interruptive function of pain. Psychol Bull.

[32] Engeln A, Schlag B, Schlag B (2008). Kompensationsstrategien im Alter. Leistungsfähigkeit und Mobilität im Alter. Mobilität und Alter.

[33] Ersek M, Cherrier MM, Overman SS (2004). The cognitive effects of opioids. Pain Manag Nurs.

[34] Fan A, Wilson KG, Acharya M, Cranney A, Buenger U, Marshall S (2012). Self-reported issues with driving in patients with chronic pain. PM R.

[35] Fastenmeier W, Gstalter H (2003). Entwicklung und Anwendung einer neuen Methodik zur Fahraufgabenanalyse. Der Fahrer im 21. Jahrhundert. Anforderungen, Anwendungen, Aspekte für Mensch-Maschine-Systeme.

[36] Ferreira DH, Boland JW, Phillips JL, Lam L, Currow DC (2018). The impact of therapeutic opioid agonists on driving-related psychomotor skills assessed by a driving simulator or an on-road driving task: a systematic review. Palliat Med.

[37] Ferreira KS, Teixeira CT, Cafaro C, Oliver GZ, Carvalho GLP, Carvalho LASD, Silva BG, Haes FBB, Ciciarelli MC (2018). Chronic migraine patients show cognitive impairment in an extende neuro-psychological assessment. Arq Neuropsiquiatr.

[38] Foley DJ, Wallace RB, Eberhard J (1995). Risk factors for motor vehicle crashes among older drivers in a rural community. J Am Geriatr Soc.

[39] Gärtner J, Elsner F, Radbruch L (2008). Einfluss von Änderungen der Opioid Tagesdosis auf fahrrelevante kognitive und psychomotorische Leistungen. Schmerz.

[40] Gaskin DJ, Richard P (2012). The economic costs of pain in the United States. J Pain.

[41] Geisser ME, Roth RS, Robinson ME (1997). Assessing depression among persons with chronic pain using the center for epidemiological studies-depression scale and the beck depression inventory: a comparative analysis. Clin J Pain.

[42] Gil-Gouveia R, Oliveira AG, Martins IP (2015). Cognitive dysfunction during migraine attacks: a study on migraine without aura. Cephalalgia.

[43] Grace GM, Nielson WR, Hopkins M (1999). Concentration and memory deficits in patients with fibromyalgia syndrome. J Clin Exp Neuropsychol.

[44] Gräcmann N, Albrecht M (2014). Begutachtungs-Leitlinien zur Kraftfahreignung.

[45] Grisart JM, Linden M, Masquelier E (2002). Controlled processes and automaticity in memory functioning in fibromyalgia patients: relation with emotional stress and hypervigilance. J Clin Exp Neuropsychol.

[46] Grisart JM, Plaghki LH (1999). Impaired selective attention in chronic pain patients. Eur J Pain.

[47] Halicka M, Vitterso AD, Proulx MJ, Bultidute HJ (2020). Neuropsychological changes in complex regional pain syndrome (CRPS). Behav Neurol.

[48] Harman K, Ruyak P (2005). Working through the pain: a controlled study of the impact of persistent pain on performing a computer task. Clin J Pain.

[49] Hart RP, Martelli MF, Zasler ND (2000). Chronic pain and neuropsychological functioning. Neuropsychol Rev.

[50] Hasenbring M, Basler HD, Franz C, Kröner-Herwig B, Rehfisch HP, Seemann H (1999). Prozesse der Chronifizierung von Schmerzen. Psychologische Schmerztherapie.

[51] Higgins DM, Martin AM, Baker DB, Vasterling JJ, Risbrough V (2018). The relationship between chronic pain and neurocognitive function: a systematic review. Clin J Pain.

[52] Hoving JL, O’Leary EF, Niere KR, Green S, Buchbinder R (2003). Validity of the neck disability index, Northwick Park neck pain questionnaire, and problem elicitation technique for measuring disability associated with whiplash-associated disorders. Pain.

[53] The International Council on Alcohol, Drugs & Traffic Safety (2012) Categorization system for medicinal drugs affecting driving performance. http://www.icadts.nl/medicinal.html. Zugegriffen: 31. Okt. 2021

[54] Jamison RN, Schein JR, Vallow S (2003). Neuropsychological effects of long-term opioid use in chronic pain patients. J Pain Symptom Manage.

[55] Johannes CB, Le TK, Zhou X, Johnston JA, Dworkin RH (2010). The prevalence of chronic pain in United States adults: results of an internet-based survey. J Pain.

[56] Jones C, Abbassian A, Trompeter A, Solan M (2010). Driving a modified car: a simple but unexploited adjunct in the management of patients with chronic right sided foot and ankle pain. Foot Ankle Surg.

[57] Jones J, McCann J, Lassere M (1991). Driving and arthritis. Rheumatology.

[58] Karp JF, Reynolds CF, Butters MA (2006). The relationship between pain and mental flexibility in older adult pain clinic patients. Pain Med.

[59] Kewman DG, Vaishampayan N, Zald D (1991). Cognitive impairment in musculoskeletal pain patients. Int J Psychiatry Med.

[60] Kurita GP, de Mattos Pimenta CA, Braga PE (2012). Cognitive function in patients with chronic pain treated with opioids: characteristics and associated factors. Acta Anaesthesiol Scand.

[61] Küst J, Dettmers C (2014). Fahreignung bei Multipler Sklerose. Nervenarzt.

[62] Küst J, Jacobs U, Karbe H (2008). Fahreignung nach neurologischen Erkrankungen: Eine quantitative Analyse. Neuro Rehabil.

[63] Lagarde E, Chastang J, Lafont S, Coeuret-Pellicer M, Chiron M (2005). Pain and pain treatment were associated with traffic accident involvement in a cohort of middle-aged workers. J Clin Epidemiol.

[64] Landro NI, Fors EA, Våpenstad LL, Holthe Ø, Stiles TC, Borchgrevink PC (2013). The extent of neurocognitive dysfunction in a multidisciplinary pain centre population. Is there a relation between reported and tested neuropsychological functioning?. Pain.

[65] Lee DM, Pendleton N, Tajar A (2010). Chronic widespread pain is associated with slower cognitive processing speed in middle-aged and older European men. Pain.

[66] Legrain V, Van Damme S, Ecclestone C, Davis KD, Seminowicz DA, Crombez G (2009). A neurocognitive model of attention to pain: behavioural and neuroimaging evidence. Pain.

[67] Louwerens JW, Gloerich ABM, De Vries G, Noordzij PC, Roszbach R (1987). The relationship between drivers’ blood alcohol concentration (BAC) and actual driving performance during high speed travel. Alcohol, drugs and traffic safety. Proceedings of the 10th International Conference on Alcohol, Drugs and Traffic Safety.

[68] Luerding R, Weigand T, Bogdahn U (2008). Working memory performance is correlated with local brain morphology in the medial frontal and anterior cingulate cortex in fibromyalgia patients: structural correlates of pain–cognition interaction. Brain.

[69] McGwin G, Sims RV, Pulley L, Roseman JM (2000). Relations among chronic medical conditions, medications, and automobile crashes in the elderly: a population-based case-control study. Am J Epidemiol.

[70] Meeus M, Van Oosterwijck J, Ickmans K (2015). Interrelationships between pain processing, cortisol and cognitive performance in chronic whiplash-associated disorders. Clin Rheumatol.

[71] Menefee LA, Frank ED, Crerand C (2004). The effects of transdermal fentanyl on driving, cognitive performance, and balance in patients with chronic nonmalignant pain conditions. Pain Med.

[72] Meyer JS, Thornby J, Crawford K (2000). Reversible cognitive decline accompanies migraine and cluster headaches. Headache.

[73] Michon JA (1979). Dealing with danger. Summary report of a workshop in the Traffic Research Center, State University, Groningen, The Nederlands.

[74] Mifflin K, Chorney J, Dick B (2016). Attention and working memory in female adolescents with chronic pain and healthy pain free female adolescents: a preliminary pilot study. Clin J Pain.

[75] Miller EK, Cohen JD (2001). An integrative theory of prefrontal cortex function. Annu Rev Neurosci.

[76] Miller RM, Kaiser RS (2018). Psychological characteristics of chronic pain: a review of curret evidence and assessment tools to enhance treatment. Curr Pain Headache Rep.

[77] Moore DJ, Keogh E, Eccleston C (2012). The interruptive effect of pain on attention. Q J Exp Psychol.

[78] Murray CB, Patel KV, Twiddy H, Sturgeon JA, Palermo TM (2012). Age differences in cognitive-affective processes in adults with chronic pain. Eur J Pain.

[79] Niemann H, Hartje W (2016). Fahreignung bei neurologischen Erkrankungen.

[80] Nilsen HK, Landrø NI, Kaasa S, Jenssen GD, Fayers P, Borchgrevink PC (2011). Driving functions in a video simulator in chronic non-malignant pain patients using and not using codeine. Eur J Pain.

[81] Nolet PS, Emary PC, Kristman VL, Murnaghan K, Zeegers MP, Freeman MD (2019). Exposure to a motor vehicle collision and the risk of future neck pain: a systematic review and meta-analysis. PM R.

[82] Ojeda B, Duenas B, Salazar A, Mico JA, Torres LM, Failde I (2018). Factors influencing cognitive impairment in neuropathic and muskuloskeletal pain and fibromyalgia. Pain Med.

[83] Okunribido OO, Shimbles SJ, Magnusson M, Pope M (2007). City bus driving and low back pain: a study of the exposures to posture demands, manual materials handling and whole-body vibration. Appl Ergon.

[84] Oosterman JM, Derksen LC, van Wijck AJ (2012). Executive and attentional & functions in chronic pain: Does performance decrease with increasing task load?. Pain Res Manag.

[85] Oosterman JM, Derksen LC, van Wijck AJ (2011). Memory functions in chronic pain: examining contributions of attention and age to test performance. Clin J Pain.

[86] Oosterman JM, Gibson SJ, Pulles WL, Veldhuijzen DS (2013). On the moderating & role of age in the relationship between pain and cognition. Eur J Pain.

[87] Park DC, Glass JM, Minear M (2001). Cognitive function in fibromyalgia patients. Arthritis Rheum.

[88] Pereira MJ, Jull GA, Treleaven JM (2008). Self-reported driving habits in subjects with persistent whiplash-associated disorder: relationship to sensorimotor and psychologic features. Arch Phys Med Rehabil.

[89] Peters B, Nilsson L, Cacciabue PC (2007). Modelling the driver in control. Modelling driver behaviour in automotive environments. Critical issues in driver interactions with intelligent transport systems.

[90] Petrovic P, Ingvar M (2002). Imaging cognitive modulation of pain processing. Pain.

[91] Poschadel S, Falkenstein M, Pappachan P, Poll E (2005). Testverfahren zur psychometrischen Leistungsprüfung der Fahreignung.

[92] Povedano M, Gascon J, Galvez R (2007). Cognitive function impairment in patients with neuropathic pain under standard conditions of care. J Pain Symptom Manage.

[93] Price JL, Drevets WC (2012). Neural circuits underlying the pathophysiology of mood disorders. Trends Cogn Sci.

[94] Pulles WL, Oosterman JM (2011). The role of neuropsychological performance in the relationship between chronic pain and functional physical impairment. Pain Med.

[95] Redelmeier DA, Zung JD, Thiruchelvam D, Tibshirani RJ (2015). Fibromyalgia and the risk of a subsequent motor vehicle crash. J Rheumatol.

[96] Reyes Del Paso G, Pulgar A, Duschek S (2012). Cognitive impairment in fibromyalgia syndrome: the impact of cardiovascular regulation, pain, emotional disorders and medication. Eur J Pain.

[97] Rodríguez-Andreu J, Ibáñez-Bosch R, Portero-Vázquez A (2009). Cognitive impairment in patients with fibromyalgia syndrome as assessed by the mini-mental state examination. BMC Musculoskelet Disord.

[98] Röijezon U, Djupsjöbacka M, Björklund M, Häger-Ross C, Grip H, Liebermann DG (2010). Kinematics of fast cervical rotations in persons with chronic neck pain: a cross-sectional and reliability study. BMC Musculoskelet Disord.

[99] Sabatowski R, Schwalen S, Rettig K, Herberg KW, Kasper SM, Radbruch L (2003). Driving ability under long-term treatment with transdermal fentanyl. J Pain Symptom Manage.

[100] Schiltenwolf M, Akbar M, Hug A (2014). Evidence of specific cognitive deficits in patients with chronic low back pain under long-term substitution treatment of opioids. Pain Phys.

[101] Schnurr RF, MacDonald MR (1995). Memory complaints in chronic pain. Clin J Pain.

[102] Shmygalev S, Dagtekin O, Gerbershagen HJ, Marcus H, Jübner M, Sabatowski R, Petzke F (2014). Assessing cognitive and psychomotor performance in patients with fibromyalgia syndrome. Pain Ther.

[103] Sjögren P, Christrup LL, Petersen MA, Hojsted J (2005). Neuropsychological assessment of chronic non-malignant pain patients treated in a multidisciplinary pain centre. Eur J Pain.

[104] Suhr JA (2003). Neuropsychological impairment in fibromyalgia: relation to depression, fatigue, and pain. J Psychosom Res.

[105] Takasaki H, Johnston V, Treleaven JM, Jull GA (2012). The Neck Pain Driving Index (NPDI) for chronic whiplash-associated disorders: development, reliability, and validity assessment. Spine J.

[106] Takasaki H, Johnston V, Treleaven JM, Pereira M, Jull G (2011). Driving with a chronic whiplash—associated disorder: a review of patients’ perspectives. Arch Phys Med Rehabil.

[107] Takasaki H, Treleaven J, Johnston V, Rakotonirainy A, Haines A, Jull G (2013). Assessment of driving-related performance in chronic whiplash using an advanced driving simulator. Accid Anal Prev.

[108] Takasaki H, Treleaven J, Johnston V, Van den Hoorn W, Rakotonirainy A, Jull G (2014). A description of neck motor performance, neck pain, fatigue, and mental effort while driving in a sample with chronic whiplash-associated disorders. Am J Phys Med Rehabil.

[109] Talusan PG, Miller CP, Save AV, Reach JS (2015). Driving reaction times in patients with foot and ankle pathology before and after image-guided injection: pain relief without improved function. Foot Ankle Spec.

[110] Tassain V, Attal N, Fletcher D (2003). Long term effects of oral sustained release morphine on neuropsychological performance in patients with chronic non-cancer pain. Pain.

[111] Vaezipour A, Andrews N, Horswill M, Johnston V, Oviedo-Trespalacios O, Delhomme P (2021). Driving behaviour in people with chronic pain–perspective of people with chronic pain and health professionals perspective of people with chronic pain and health professionals.

[112] Vaezipour A, Oviedo-Trespalacios O, Horswill M, Rod JE, Andrews N, Johnston V, Delhomme P (2022). Impact of chronic pain on driving behaviour: a systematic review. Pain.

[113] Veldhuijzen DS, van Wijck AJM, Wille F, Verster JC, Kenemans JL, Kalkman CJ, Olivier B, Volkerts ER (2006). Effect of chronic nonmalignant pain on highway driving performance. Pain.

[114] Veldhuijzen DS, Sondaal SF, Oosterman JM (2012). Intact cognitive inhibition in & patients with fibromyalgia but evidence of declined processing speed. J Pain.

[115] Verdejo-García A, López-Torrecillas F, Calandre EP, Delgado-Rodríguez A, Bechara A (2009). Executive function and decision-making in women with fibromyalgia. Arch Clin Neuropsychol.

[116] Villemure C, Bushnell MC (2002). Cognitive modulation of pain: How do attention and emotion influence pain processing?. Pain.

[117] Weiner DK, Rudy TE, Morrow L (2006). The relationship between pain, neuropsychological performance, and physical function in community-dwelling older adults with chronic low back pain. Pain Med.

