# Supplementary material for: Cognition and driving ability in chronic pain syndrome
Source: Nervenarzt. 2022 Sep 28;94(4):335–43. [Article in German] doi: 10.1007/s00115-022-01387-y (PMC10104908; doi:10.1007/s00115-022-01387-y)
Supplement: Supplementary file 1 [file 115_2022_1387_MOESM1_ESM.pdf]

eTab. 1 Studienlage Chronisches Schmerzsyndrom und Fahrverhalten
CS = Chronischer Schmerz, KG = Kontrollgruppe

**Autoren**<br>**Land**	**Stichprobe**<br>**Alter**	**Methode**	**Neurokognitive Domänen/ Verfahren**<br>**Ergebnisse**	**Psychosoziale Verfahren**<br>**Ergebnisse**	**Fahrverhalten**<br>**Ergebnisse**	**Einteilung nach Michon**<br>Operational (I)<br>Taktisch (II)<br>Strategisch (III)
Bell et al., 2020 [11]<br>USA	CS (Rücken)<br>n = 308,<br>KG n = 813,<br>Alter: +55	Selbstauskunft, Kognitive Tests<br>Längsschnittstudie über 15 Jahre	Geteilte Aufmerksamkeit: Useful Field of View (UFOV) Subtest 2, Verarbeitungsgeschwindigkeit: TMT A, Kognitive Umstellungsfähigkeit: TMT B, Visuospatiale Wahrnehmung: Motor Visual Perception Task (MVPT)<br>Zusammenhänge zwischen MVPT und Autounfällen		Höhere Wahrscheinlichkeit für Autounfälle bei CS (Rücken)	II (z.B. Einschätzen von Abständen zwischen Fahrzeugen)
Benyamina et al., 2018 [12]<br>Kanada	CS (Rücken)<br>n = 621,<br>Akut (Rücken)<br>n = 899,<br>KG n = 688,<br>Alter: 0 - +55<br>Polizeibeamte	Selbstauskunft		Perceived Stress Scale (PSS), Patient Health Questionnaire (PHQ-2), Generalized Anxiety Disorder scale (GAD-2), Chronic Pain Sleep Inventory (CPSI)<br>Unterschiede in der Schlafqualität zwischen den Gruppen	Geringerer Komfort beim Autofahren bei CS	
Byas-Smith et al., 2005 [21]<br>USA	CS (Rücken, Nacken, Kopf)<br>n = 11,<br>Alter: 46.5 (6.9)<br>CS (Rücken, Nacken, Kopf, Opioide) n = 21,<br>Alter: 47.7 (10.9)<br>KG n = 50,<br>Alter: 42.6 (9.1)	Selbstauskunft, Kognitive Tests, Autofahrt	Aufmerksamkeit: Test of Variables of Attention (TOVA)<br>Verarbeitungsgeschwindigkeit: Zeichen Symbol Test (ZST).<br>KG höhere ZST Werte (Keine Zusammenhänge mehr nach Kontrolle von Alter und Bildung)	Visuelle Analog Skala (VAS)	Keine Gruppenunterschiede	

**Autoren**<br>**Land**	**Stichprobe**<br>**Alter**	**Methode**	**Neurokognitive Domänen/ Verfahren**<br>**Ergebnisse**	**Psychosoziale Verfahren**<br>**Ergebnisse**	**Fahrverhalten**<br>**Ergebnisse**	**Einteilung nach Michon**
Fan et al., 2012 [34]<br>Kanada	CS (Nacken, Rücken) n = 223,<br>Alter: 49.9 (10.4)<br>CS Fahrer<br>n = 176,<br>Alter: 49.3 (10.1)<br>CS keine Fahrer<br>n = 41,<br>Alter: 52.5 (11.9)<br>CS (k.A) n = 6	Selbstaus-kunft		Numerische Rating Skala (NRS), Pain Disability Index (PDI), Patient Health Questionnaire<br>Gruppenunterschiede im PDI	CS 70% Einschränkungen beim Fahren davon Schmerz (88,6%), Fatigue (50,6%), eingeschränkte Mobilität (48,3%), Schwächegefühl (19,4%), keine Schwierigkeiten (6%). 6% berichteten Unfälle im letzten Jahr. 56% stellten Autofahren wegen Schmerzen ein.	
Foley et al., 1995 [38]<br>USA	n = 1791,<br>Alter: +68.<br>Iowa Department of Motor Vehicles	Selbstaus-kunft	Short portable mental status questionnaire (SPMSQ), Gedächtnis: 20 Wörter Gedächtnistest<br>Zusammenhänge zwischen schlechten Gedächtnisleistungen und Autounfällen	Center for Epidemiologic Studies Depression (CES-D)<br>Zusammenhänge zwischen depressiven Symptomen und Autounfällen	Erhöhtes Unfallrisiko bei Menschen mit Rückenschmerzen	
Hoving et al., 2003 [52]<br>Australien	CS (Nacken, Kopf, Schulter, Arm, Rücken)<br>n = 71,<br>Alter: 40.1 (14.3)	Selbstaus-kunft		Neck Pain Questionnaire (NPQ), Neck Disability Index (NDI), Problem elicitation technique (PET)<br>PET = 7,7 Probleme: Arbeit für wenig Lohn (52.1%), Fatigue (50.7%), Sport (47.9%), Depression (43.7%), Freundschaften (33.8%), Schlaf (31.0%), Frustration (31.0%), Ärger (28.2%).	Berichtete Schwierigkeiten beim Autofahren (43,7%)	

Autoren<br>Land	Stichprobe<br>Alter	Methode	Neurokognitive Domänen/ Verfahren<br>Ergebnisse	Psychosoziale Verfahren<br>Ergebnisse	Fahrverhalten<br>Ergebnisse	Einteilung nach Michon
Jones et al., 1991 [57]<br>Neuseeland	CS (Rheumatoide Arthritis, Osteoarthritis, Fibromyalgie)<br>n = 94,<br>Alter: k.A.	Selbstaus-kunft, Autofahrt			82% als sichere Fahrer eingeschätzt. Empfehlung Modifikation Fahrzeug an Einschränkungen.	
Jones et al., 2010 [56]<br>UK	CS (Fuß, Knöchel)<br>n = 20,<br>Alter: 62.7	Selbstaus-kunft, Telefon-interview			Eingeschränktes Fahren durch Knöchel- oder Fußschmerzen.<br>5% nicht mehr fähig zum Autofahren.<br>Empfehlung Umbau des Fahrzeuges.<br>Mögliche Umbaumaßnahme weitgehend unbekannt bei Behandlern.	
Lagarde et al. 2005 [63]<br>Frankreich	n = 13843,<br>Alter: +35	Selbstaus-kunft			Erhöhtes Risiko für Autounfälle durch Schmerz und Schmerzbehandlung.<br>Zusammenhang Verkehrsunfälle und Zahn- und Zahnfleischprobleme bei Männern, Nierenkolik und Nierensteine bei Frauen.	
McGwin et al. 2000 [69]<br>USA	n = 901,<br>Alter: +65	Selbstaus-kunft, Telefon-interview	SPMSQ		Erhöhtes Risiko für Autounfälle bei Frauen mit Arthritis.	

Autoren<br>Land	Stichprobe<br>Alter	Methode	Neurokognitive Domänen/<br>Verfahren<br>Ergebnisse	Psychosoziale Verfahren<br>Ergebnisse	Fahrverhalten<br>Ergebnisse	Einteilung<br>nach Michon
Nilsen et al. 2011 [80]<br>Norwegen	CS n = 20,<br>Alter: 42.4 (8.0)<br>CS (keine Opioide)<br>n = 20,<br>Alter: 43.2 (6.2)<br>KG n = 20,<br>Alter: 37.7 (10.0)	Fahr-<br>simulator		EORTC QLQ-C30,<br>Big Five Inventory (BFI).<br>Gruppenunterschiede in allen Skalen des EORTC QLQ-C30 sowie Extraversion und Neurotizismus	Gruppen-<br>unterschiede Reaktionszeit und Beachtung von Verkehrsschildern:<br>CS > KG.<br>Keine Unterschiede zwischen CS mit und ohne Opioide in Bezug auf Fahrverhalten	
Okunribido et al., 2006 [83]<br>UK	CS (Rücken)<br>n = 61,<br>Alter: 19-64<br>Busfahrer	Selbstaus-<br>kunft,<br>Busfahrt		Kein Einfluss des Schmerzes auf die Teilnahme an sozialen Aktivitäten	Wenig Einfluss des Rückenschmerzes auf die Arbeit.<br>20% vermeiden Autofahren aufgrund von Schmerz.	
Pereira et al., 2008 [88]<br>Australien	CS (Schleudertrau-<br>mata)<br>n = 30,<br>Alter:<br>33,8 (9,4)<br>KG n = 30,<br>Alter: 25.6 (5.1)	Selbstaus-<br>kunft,<br>Messung Rotation HWS		NDI, General Health Questionnaire (GHQ-28), Impact of Events Scale Revised (IES- R), Tampa Scale for Kinesiophobia,<br>Gruppenunterschiede GHQ-28, NDI, IES-R	Mehr berichtete Schwierigkeiten beim Fahren bei CS.<br>27% benötigten Unterstützung durch Mitfahrer, 23% Angabe, dass sie Fahren reduzieren sollten.	
Röijezon et al., 2010 [98]<br>Schweden	CS (Nacken)<br>n1 = 16,<br>Alter: 45 (10)<br>KG n1 = 16,<br>Alter: 48 (7),<br>CS n2 = 102,<br>Alter: 47 (10),<br>KG n2 = 33,<br>Alter: 51 (9)<br>Nur Frauen	Selbstaus-<br>kunft,<br>Messung Rotation HWS		NRS, Short Form Health Survey (SF-36), NDI, Disability Arm Shoulder Hand Questionnaire (DASH), Tampa Scale of Kinesiophobia (TSK)	CS selbstberichtete Einschränkungen beim Autofahren	

Autoren Land	Stichprobe Alter	Methode	Neurokognitive Domänen/ Verfahren Ergebnisse	Psychosoziale Verfahren Ergebnisse	Fahrverhalten Ergebnisse	Einteilung nach Michon
Redelmeier et al., 2015 [95] Kanada	CS (Fibromyalgie) n = 137631, Alter: 0 - +60	Retro-spektive Daten-analyse			Fibromyalgie ist mit einer erhöhten Wahrscheinlichkeit eines Unfallgeschehens verbunden	
Shmygalev et al. 2014 [102] Deutschland	CS (Fibromyalgie) n = 43, Alter: 38-75 KG n = 129, Alter: 38- 75 Nur Frauen	Selbstaus-kunft, Kognitive Tests	Aufmerksamkeit: Cognitrone (COG), Belastbarkeit: Determinationstest (DT), Motorische Koordination: 2-Hand, Vigilanz (VIG). Längere Reaktionszeit im DT bei CS	VAS, Beck Depression Inventory (BDI), SF-36, McGill Pain Questionnaire (SF-MPQ), Brief Pain Inventory (BPI), Brief Fatigue Inventory (BFI), State-Trait Anxiety Inventory (STAI) Schmerz, jüngeres Alter, Depression, Angst, Fatigue, eingeschränkte motorische Koordination in Zusammenhang mit subjektiver Wahrnehmung kognitiver Dysfunktion	Keine Unterschiede in der Kraftfahreignung	
Takasaki et al., 2011 [106] Australien	CS (Schleudertrauma) n = 33. n_1 = 24, Alter: 36.0 (9.9), n_2 = 9, Alter: 33.9 (9.4)	Selbstaus-kunft, Interview		NDI, Visual analog scale, Driving Habit Questionnaire 54% Angst/ Nervosität beim Autofahren 50% vorsichtigeres Fahren	73% berichteten Einschränkungen beim Autofahren: Blinde Flecken (taktisch), längeres Fahren (strategisch), rückwärts einparken (operational), Rumpfrotation. 17% Vermeidung bestimmter Situationen	I, II, III
Takasaki et al., 2012 [105] Australien	CS (Schleudertrauma) n = 87, Alter: 38.4 (11.5)	Selbstaus-kunft		NDI, NRS 20% selbstberichtete Kopf-/ Nackenschmerzen, Schmerzen bei der schnellen Nackenrotation, Stress, Angst	Einschränkungen bei Fahrten länger als 1 Stunde (strategisch), blinde Flecken (taktisch) und Wendemanöver (operational)	I, II, III

**Autoren Land**	**Stichprobe Alter**	**Methode**	**Neurokognitive Domänen/ Verfahren Ergebnisse**	**Psychosoziale Verfahren Ergebnisse**	**Fahrverhalten Ergebnisse**	**Einteilung nach Michon**
Takasaki et al., 2013a [107] Australien	CS (Schleudertrauma) n = 40, Alter: 34.7 (11.4)	Selbstauskunft, Messung Nackenrotation	Fatigue Severity Scale, Modified-Perceived Deficit Questionnaire, Verarbeitungsgeschwindigkeit: TMT A, Kognitive Umstellungsfähigkeit: TMT B Zusammenhänge zwischen kognitiver Domäne und strategischer/ operationaler Ebene	NDI, General Health Questionnaire (GHQ-28), IES-R, Fear of Activity Scale-Cervical (19-item PEAetS-C), Fear section of the Travel Phobia Questionnaire (F-TPQ) Zusammenhänge zwischen physischer Domäne und taktischem Level	Zusammenhang zwischen Symptomdauer und Einschränkungen auf strategischer und taktischer Ebene.	I (z.B. abruptes Bremsen), II (z.B. längere Fahrten), III
Takasaki et al., 2013b [108] Australien	CS (Schleudertrauma) n = 17, Alter: 35.1 (12.0) KG n = 26, Alter: 36.7 (10.4)	Selbstauskunft, Fahrsimulator	Reaktion auf roten Punkt in Rückspiegel durch Blinken Keine Gruppenunterschiede bei Reaktionszeiten	NDI, Modified Motion Sickness Assessment Questionnaire (M-MSAQ)	Keine Gruppenunterschiede im Fahrsimulator	
Takasaki et al., 2014 [109] Australien	CS (Schleudertrauma) n = 14, Alter: 33.4 (10.8). KG n = 14, Alter: 35.4 (10.7)	Selbstauskunft, Messung Nackenrotation Fahrsimulator	Rating Scale Mental Effort (RSME). Größerer mentaler Aufwand bei CS	NDI, Neck Pain Driving Index (NPDI), NRS, VAS-fatigue, Modified Motion Sickness Assessment Questionnaire (M-MSAQ), Dizziness Handicap Inventory short form, Modified Perceived Deficit Questionnaire, 28-item General Health Questionnaire, IES-R, Fear section of the Travel Phobia Questionnaire	Keine Gruppenunterschiede im Fahrsimulator. Korrelation von selbstberichteten Einschränkungen beim Fahren mit Intensität Schmerz, Maximalgeschwindigkeit, Nackenrotation, Fatigue	

Autoren<br>Land	Stichprobe<br>Alter	Methode	Neurokognitive Domänen/ Verfahren<br>Ergebnisse	Psychosoziale Verfahren<br>Ergebnisse	Fahrverhalten<br>Ergebnisse	Einteilung nach Michon
Talusan et al., 2015 [110]<br>USA	CS (Fuß, Knöchel)<br>n = 26,<br>Alter: k.A.<br>KG n = 11,<br>Alter: k.A.	Simulation Messung Reaktions-zeit vor und nach Schmerz-injektion in Fuß/ Knö-chel			Gruppenunterschiede bei Bremsreaktion	
Thiese et al., 2015 [112]<br>USA	n = 797,<br>Alter: 47.2 (10.5)<br>LKW-Fahrer	Selbstaus-kunft			Zusammenhang zwischen Rückenschmerzen und Unfallhäufigkeit (35,4%).	
Veldhuijzen et al. 2006 [116]<br>Niederlande	CS (Rücken, Knie)<br>n = 14,<br>Alter: 46.9 (8.5)<br>KG n = 14,<br>Alter: 50.0 (7.5)	Selbstaus-kunft, Kognitive Tests, Autofahrt	Belastbarkeit: Tracking Test, Gedächtnis und Reaktionsge-schwindigkeit: Sternberg memory scanning test, Geteilte Aufmerksamkeit: Divided attention test<br>Keine Gruppenunterschiede	BDI, Spielberger State-Trait Anxiety scales (STAI), SF-36, Dutch reading test for adults (NLV), VAS, McGill Pain Questionnaire (MPQ), Coping with Pain Questionnaire (CPQ), Coping Strategy Questionnaire (CSQ)<br>Gruppenunterschiede im SF-36	Standard deviation of lateral position (SDLP):<br>CS > KG.<br>Erhöhtes Risiko für schlechtere Leistungen beim Autofahren.<br>Subjektive Fahrqualität<br>CS < KG.	II
