# Supplementary material for: Cognition and driving ability in chronic pain syndrome
Source: Nervenarzt. 2022 Sep 28;94(4):335–43. [Article in German] doi: 10.1007/s00115-022-01387-y (PMC10104908; doi:10.1007/s00115-022-01387-y)
Supplement: Supplementary file 2 [file 115_2022_1387_MOESM2_ESM.pdf]

11. Bell T, Pope C, Fazeli P, Crowe M, Ball K (2020) The association of persistent low back pain with older adult falls and collisions: a longitudinal analysis. Journal of applied gerontology 0733464820966517

12. Benyamina Douma N, Côté C, Lacasse A (2018) Occupational and Ergonomic Factors Associated with Low Back Pain Among Car-patrol Police Officers. The Clinical Journal of Pain 34(10):960¬966

21. Byas-Smith MG, Chapman SL, Reed B, Cotsonis G (2005) The effect of opioids on driving and psychomotor performance in patients with chronic pain. The Clinical journal of pain21(4):345-352

34. Fan A, Wilson KG, Acharya M, Cranney A, Buenger U, Marshall S (2012) Self-reported issues with driving in patients with chronic pain. PM&R4:87–95

38. Foley DJ, Wallace RB, Eberhard J (1995) Risk factors for motor vehicle crashes among older drivers in a rural community. Journal of the American Geriatrics Society43(7):776- 781

52. Hoving JL, O'Leary EF, Niere KR, Green S, Buchbinder R (2003) Validity of the neck disability index, Northwick Park neck pain questionnaire, and problem elicitation technique for measuring disability associated with whiplash-associated disorders. Pain102(3):273-281

56. Jones C, Abbassian A, Trompeter A, Solan M (2010) Driving a modified car: A simple but unexploited adjunct in the management of patients with chronic right sided foot and ankle pain. Foot and ankle surgery16(4):170-173

57. Jones J, McCann J, Lassere M (1991) Driving and arthritis. Rheumatology 30(5):361-364

63. Lagarde E, Chastang J, Lafont S, Coeuret-Pellicer M, Chiron M (2005) Pain and pain treatment were associated with traffic accident involvement in a cohort of middle-aged workers. JClin Epidemiol 58(5):524–531

69. McGwin G, Sims RV, Pulley L, Roseman JM (2000) Relations among chronic medical conditions, medications, and automobile crashes in the elderly: a population-based case-control study. Am J Epidemiol; 152:424–31

80. Nilsen HK, Landrø NI, Kaasa S, Jenssen GD, Fayers P, Borchgrevink PC. (2011) Driving functions in a video simulator in chronic non-malignant pain patients using and not using codeine. Eur J Pain15 409–15

83. Okunribido OO, Shimbles SJ, Magnusson M, Pope M (2007) City bus driving and low back pain: a study of the exposures to posture demands, manual materials handling and whole-body vibration. Applied ergonomics38(1):29-38

88. Pereira MJ, Jull GA, Treleaven JM (2008) Self-reported driving habits in subjects with persistent whiplash-associated disorder: relationship to sensorimotor and psychologic features. Archives of physical medicine and rehabilitation89(6):1097-1102

95. Redelmeier DA, Zung JD, Thiruchelvam D, Tibshirani RJ (2015) Fibromyalgia and the risk of a subsequent motor vehicle crash. The Journal of rheumatology42(8):1502-1510

98. Röijezon U, Djupsjöbacka M, Björklund M, Häger-Ross C, Grip H, Liebermann DG (2010) Kinematics of fast cervical rotations in persons with chronic neck pain: a cross-sectional and reliability study. BMC musculoskeletal disorders11(1):1-10

102. Shmygalev S, Dagtekin O, Gerbershagen HJ, Marcus H, Jübner M, Sabatowski R, Petzke F (2014) Assessing cognitive and psychomotor performance in patients with fibromyalgia syndrome. Pain and therapy3(2):85-101

105. Takasaki H, Johnston V, Treleaven JM, Jull GA (2012) The Neck Pain Driving Index (NPDI) for chronic whiplash-associated disorders: development, reliability, and validity assessment. The Spine Journal12(10):912-920. e911

106. Takasaki H, Johnston V, Treleaven JM, Pereira M, Jull G (2011) Driving with a chronic whiplash-associated disorder: a review of patients' perspectives. Archives of physical medicine and rehabilitation 92(1):106-110

107. Takasaki H, Treleaven J, Johnston V, Jull G (2013a) Contributions of physical and cognitive impairments to self-reported driving difficulty in chronic whiplash-associated disorders. Spine 38(18):1554-1560

108. Takasaki H, Treleaven J, Johnston V, Rakotonirainy A, Haines A, Jull G (2013) Assessment of driving-related performance in chronic whiplash using an advanced driving simulator. Accident Analysis & Prevention60:5-14

109. Takasaki H, Treleaven J, Johnston V, Van den Hoorn W, Rakotonirainy A, Jull G (2014) A description of neck motor performance, neck pain, fatigue, and mental effort while driving in a sample with chronic whiplash-associated disorders. American journal of physical medicine & rehabilitation93(8):665-674

110. Talusan PG, Miller CP, Save AV, Reach Jr JS (2015) Driving reaction times in patients with foot and ankle pathology before and after image-guided injection: pain relief without improved function. Foot & ankle specialist 8(2):107-111

112. Thiese MS, Ott U, Robbins R, Effiong A, Murtaugh M, Lemke MR, Deckow-Schaefer G, Kapellusch J, Wood E, Passey D (2015) Factors associated with truck crashes in a large cross section of commercial motor vehicle drivers. Journal of occupational and environmental medicine57(10):1098-1106

116. Veldhuijzen DS, vanWijck AJM, Wille F, Verster JC, Kenemans JL, Kalkman CJ, Olivier B, Volkerts ER (2006) Effect of chronic nonmalignant pain on highway driving performance. Pain122 28-35
